# Supplementary material for: Single cell resolution 3D imaging and segmentation within intact live tissues
Source: Npj Imaging. 2025 Sep 3;3:40. doi: 10.1038/s44303-025-00099-7 (PMC12408837; doi:10.1038/s44303-025-00099-7)
Supplement: Supplementary file 1 — Supplementary Information [file 44303_2025_99_MOESM1_ESM.docx]

# Supplementary Information

### Supplementary Note 1: Pre-processing: image restoration

Deconvolution is a powerful approach that aims to restore an image degraded by blurring and noise to improve image quality while preserving the structures of interest. We tested two deconvolution/deblurring strategies to see whether they could improve image quality and segmentation outcomes.

#### Deconvolution with a measured PSF

For the most accurate results, an experimental point spread function (PSF) needs to be measured using fluorescent beads on the same microscope, with the same settings used to acquire the images to be segmented:

1. Prepare a sample of fluorescent beads and image it using the same microscopy settings used for the samples to be segmented. For a detailed example protocol, see <https://www.protocols.io/view/monitoring-the-point-spread-function-for-quality-bp2l61ww1vqe/v1>
2. In Huygens, distil a PSF using the beads image. For detailed instructions, see https://svi.nl/Huygens-PSF-Distiller
3. In Huygens, deconvolve the samples to be segmented using the measured PSF.
4. Once you have obtained a deconvolved image, you can drag and drop it into Cellpose. In Cellpose, segment the image using the steps explained in the *“Initial segmentation: Cellpose”* section.

** We used Huygens software but many alternative deconvolution software are available.

Overall, while image restoration improved the visual appearance of the images tested, this did not correspond to improved segmentation results (see Supplementary Figure 2). In particular, running the segmentation pipeline on deconvolved images resulted in highly fragmented labels. We did however find that acquiring images of beads was a useful reference for checking the PSF quality and helped us fine-tune some microscopy parameters, for example the objective correction collar. For this purpose, we compared the full width half maximum (FWHM) of the PSF as estimated in Huygens and aimed for the smallest and most uniform FWHM.


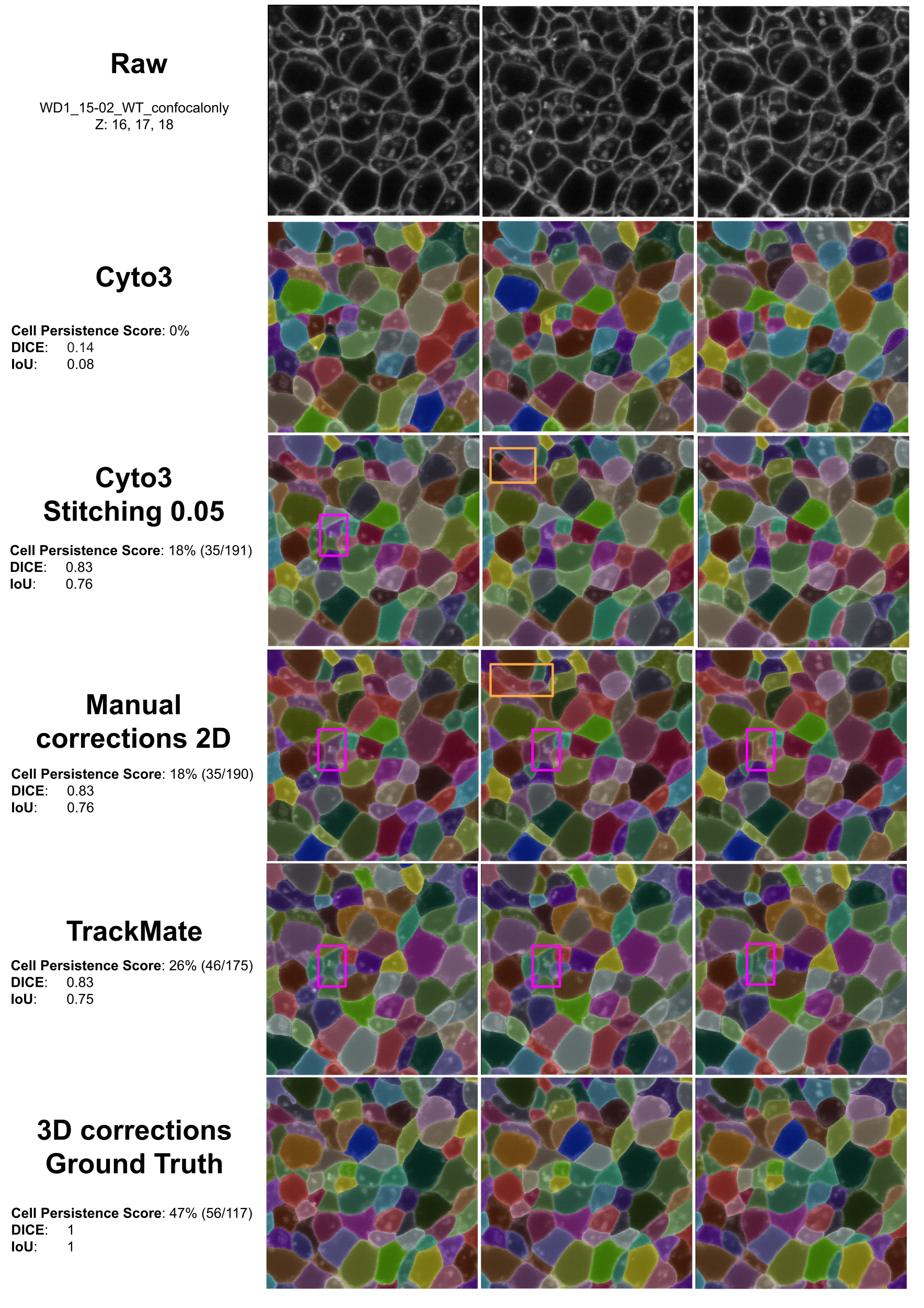


**Supplementary Figure 1**. Outcome and quality of the segmentation after each step of the protocol. First row, we display three consecutive slices from an image of our dataset. Cyto3 corresponds to the segmented images using Cyto3 model without stitching. Cyto3 with Stitching 0.05 improves in all the scores the quality of the segmentations (Cell Persistence score, Dice, and IoU). Manual corrections were performed on two different cells in Manual Corrections 2D. TrackMate was used to improve the stitching of the cells in 3D. And 3D corrections ground truth (GT) corresponds with the GT dataset used to improve the pre-trained model. Orange box: Cell that was not fully segmented, corrected after 2D manual corrections. Pink box: Fragmented cell after ‘Cyto3 with stitching’, corrected in 2D with manual corrections, and corrected in 3D using TrackMate. We can see the cell correctly segmented throughout the selected z slices in 3D and 2D. Cell Persistence score was calculated as explained in section 4.3. DICE score computes two multiplied by the intersection divided by the total number of pixels corresponding to a given identifier in both GT and segmented. IoU (Intersection-over-Union) refers to the area of overlap of the predicted and GT cell (intersection) over the area that belong to both cells (union). Note how for some sets of images (manual corrections 2D vs TrackMate) the Cell Persistence score captures an improvement in the segmentation, but the other metrics don’t.


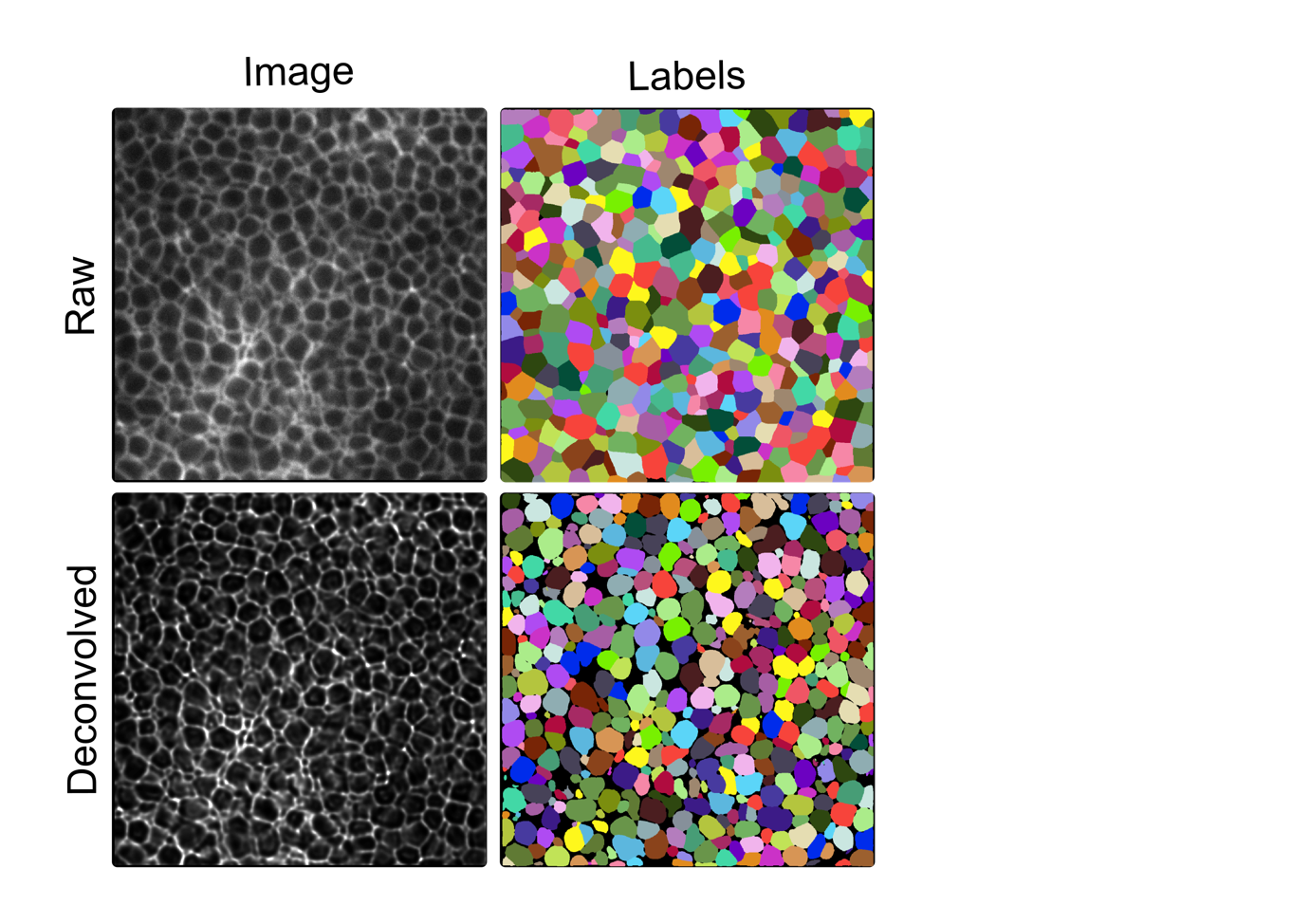


**Supplementary Figure 2.** Comparison of Cellpose segmentation on raw vs deconvolved data. Top row: example raw slice from an image in our dataset and corresponding labels predicted by Cellpose. Bottom row: same dataset, after deconvolution in Huygens using an experimentally distilled PSF. Cellpose settings for both images were the same: cyto3 model, diameter=50, stitch_threshold=0.05.
